# Associations among Caesarean Section Birth, Post-Traumatic Stress, and Postpartum Depression Symptoms

**DOI:** 10.3390/ijerph19084900

**Published:** 2022-04-18

**Authors:** Marie-Andrée Grisbrook, Deborah Dewey, Colleen Cuthbert, Sheila McDonald, Henry Ntanda, Gerald F. Giesbrecht, Nicole Letourneau

**Affiliations:** 1 Faculty of Nursing, University of Calgary, Calgary, AB T2N 4V8, Canada; marie.grisbrook@ucalgary.ca (M.-A.G.); cacuthbe@ucalgary.ca (C.C.); 2 Alberta Children’s Hospital Research Institute, Owerko Centre, Calgary, AB T2N 1N4, Canada; dmdewey@ucalgary.ca (D.D.); hentry.ntanda@ucalgary.ca (H.N.); ggiesbre@ucalgary.ca (G.F.G.); 3 Department of Community Health Sciences, Cumming School of Medicine, University of Calgary, Calgary, AB T2N 4N1, Canada; sheila.mcdonald@albertahealthservices.ca; 4 Department of Pediatrics, Cumming School of Medicine, University of Calgary, Calgary, AB T2N 4N1, Canada; 5 Hotchkiss Brain Institute, Cumming School of Medicine, University of Calgary, Calgary, AB T2N 4N1, Canada; 6 Department of Oncology, Cumming School of Medicine, University of Calgary, Calgary, AB T2N 4N1, Canada; 7 Department of Psychiatry, Cumming School of Medicine, University of Calgary, Calgary, AB T2N 4N1, Canada

**Keywords:** caesarean section, postpartum depression, post-traumatic stress disorder, mode of delivery, maternal mental health, conditional process modeling, APrON study

## Abstract

Caesarean section (C-section) deliveries account for nearly 30% of births annually with emergency C-sections accounting for 7–9% of all births. Studies have linked C-sections to postpartum depression (PPD). PPD is linked to reduced quality of parent-child interaction, and adverse effects on maternal and child health. New mothers’ perceptions of more negative childbirth experiences, such as unplanned/emergency C-sections, are linked to post-traumatic stress disorder (PTSD), which in turn is related to PPD. Our objectives were to determine: (1) the association between C-section type (unplanned/emergency vs. planned) and PPD symptoms, and (2) if postnatal PTSD symptoms mediate this association. Employing secondary analysis of prospectively collected data from 354 mother-child dyads between 2009 and 2013 from the Alberta Pregnancy Outcomes and Nutrition (APrON) study, conditional process modeling was employed. The Edinburgh Postnatal Depression Scale (EPDS) and the Psychiatric Diagnostic Screening Questionnaire (PDSQ) were administered at three months postpartum, to assess for postpartum depressive and post-traumatic stress symptoms. The direct effect of emergency C-section on PPD symptoms was non-significant in adjusted and non-adjusted models; however, the indirect effect of emergency C-section on PPD symptoms with PTSD symptoms as a mediator was significant after controlling for prenatal depression symptoms, social support, and SES (β = 0.17 (*SE* = 0.11), 95% CI [0.03, 0.42]). This suggests that mothers who experienced an emergency or unplanned C-section had increased PTSD scores of nearly half a point (0.47) compared to mothers who underwent a planned C-section, even after adjustment. Overall, emergency C-section was indirectly associated with PPD symptoms, through PTSD symptoms. Findings suggest that PTSD symptoms may be a mechanism through which emergency C-sections are associated with the development of PPD symptoms.

## 1. Introduction

Seventeen percent of mothers suffer from symptoms of postpartum depression (PPD) in the first year after delivery [1], which includes feelings of sadness, worry, and fatigue. Associated with negative maternal physical and psychological health, PPD also negatively impacts women’s relationships with their partners, and their children’s health and well-being [2]. PPD has been linked to birth experiences and obstetric factors such as pre-eclampsia, premature delivery, and caesarean section (C-section) [3,4]. C-section deliveries are common, accounting for up to 32% of all deliveries throughout North America [5]. A mother’s experience of an elective versus emergency C-section can play a role in the development of post-traumatic stress disorder (PTSD); among women with PTSD, 65% also present with PPD [6]. With C-sections representing 1/3 of all North American deliveries annually, C-section deliveries may impact women’s adjustment during the postpartum period and contribute to an increased risk of PTSD and PPD symptoms. Given the suffering associated with PPD and those women experiencing PPD incur greater health care costs [7,8], understanding the association between C-section birth and PPD may help guide interventions to address both the psychosocial impact on families and the economic impacts on health care.

## 2. Theoretical Background and Hypotheses

### 2.1. Postpartum Depression

PPD also referred to as peripartum depression [9], is classified as a major depressive disorder (MDD) with a peripartum onset modifier and is typically diagnosed during pregnancy or in the four weeks following delivery [10]. PPD has a global prevalence rate of 17.7% and has the greatest risk of onset in the first three months postpartum [11,12]. Risk factors associated with PPD are a history of depression, early gestational age, low-birthweight infants, low income, and maternal pregnancy conditions such as gestational diabetes [11,12,13]. The Diagnostic and Statistical Manual of Mental Disorders (DSM)-IV identifies that 50% of postpartum depressive episodes occur prior to childbirth and thus perinatal and postpartum depressive episodes are referred to as peripartum episodes [10]. The DSM-IV diagnostic criteria for MDD align with the International Classification of Diseases (ICD)-10 codes and are identified as 296 [10]. Many studies have examined PPD symptoms, rather than MDD diagnosis [14,15,16], using standardized scales such as the Edinburgh Postnatal Depression Scale (EPDS) [17]. PPD symptoms have severe implications for mothers, as suicides account for up to 20% of postpartum deaths [18]. Mothers with PPD symptoms have higher utilization of emergency departments and counseling services, resulting in 90% greater healthcare expenditures than non-depressed women [7,8]. A higher number of PPD symptoms has also been associated with adverse parent-child interactions [19] and decreased maternal engagement and responsiveness with their infants [19]. The long-term health outcomes for children associated with disturbed mother-infant interactions include social, emotional, cognitive, and physical development issues [19,20]. Considering the serious and long-term health outcomes for both mothers and their infants, it is crucial that factors that contribute to PPD symptoms among new mothers be identified.

### 2.2. C-Section Births

C-sections account for 28–32% of births in the developed world [21,22,23]. They are classified into two categories described as either “planned” or “emergency”. A planned or elective C-section occurs before the onset of labor and is generally scheduled between 37–39 weeks gestational age [24,25]. This type of C-section usually occurs because of a medical condition, complication, or at the clinician’s or mother’s discretion [25]. An emergency C-section typically occurs after labor has started and results from a serious complication of pregnancy or labor [24,25]. C-sections are further defined as primary and secondary with primary representing women who are having a C-section for the first time [26]. The role of a C-section delivery and the implication of these delivery methods on the mother’s health must be considered to guide the care of families.

### 2.3. C-Section Births and PPD Symptoms

PPD has been associated with C-section birth. Two population cohort studies with sample sizes ranging from nearly 400,000 to more than a million women showed that C-section birth increased the odds of women developing PPD, according to the ICD-10 diagnostic codes for depression; OR = 1.33 (95% CI, 1.28–1.38) [27]; IRR, 1.32, (95% CI, 1.13–1.53) [11]. Multiple studies have identified a positive association between C-section delivery and PPD diagnosis and symptoms, using ICD-10 diagnostic codes and screening tools such as the EPDS using a cut-off of >9 indicating a need for further assessment for depression or the Center for Epidemiologic Studies Depression Scale (CES-D) with scores ≥ 16 classified as high risk for depression [11,27,28,29,30,31]. Although two studies [28,30] identified an association between C-section delivery and PPD, these compared vaginal to C-section delivery and primary versus secondary C-section, respectively; they did not differentiate between C-section types.

In contrast, one study [32] did not find an association between mode of birth and postnatal psychological morbidity when comparing vaginal delivery to instrumental or C-section delivery, and another study [16] did not find an association between mode of delivery (i.e., vaginal versus C-section) and PPD. However, neither of these studies differentiated between C-section types when comparing vaginal versus C-section delivery. A systematic review of 24 studies [14] evaluating the link between C-section and PPD, found that 15 of the studies showed no association, four reported mixed results and only five found that C-section birth predicted PPD. A variety of factors may impact a woman’s experience of a C-section such as planned versus emergency, general anesthesia versus an epidural, amount of information provided, and the amount of control experienced [14], which could account for the variability in results among the studies included. It is hypothesized that C-sections operate as a risk factor for PPD if women are already vulnerable to PPD and that the impact of a C-section may depend on the context in which a C-section occurs (e.g., unplanned/emergency vs. planned) [14]. It is possible that the mixed findings of previous research are due to an inability to distinguish between planned and emergency C-section types.

### 2.4. Birth Experiences and PTSD

A systematic review reported a significant association between women’s perspectives of their birth experience and PPD [3]. Further, emergency C-section was identified specifically as the most influential factor associated with negative perceptions of the birth experience at one year postpartum [33,34]. A study of 326 postpartum women found that an emergency C-section was associated with increased PTSD symptoms as compared to an elective C-section or normal vaginal delivery [35].

PTSD affects 4–17% of mothers [36] and like PPD, places children at risk for altered mother-child interaction [37], and poor social-emotional development [38]. PTSD is a stress-related disorder triggered by exposure to actual or threatened death or serious injury, characterized by symptoms of re-experiencing the traumatic event, avoidance, negative cognitions and mood, hyper-vigilance, aggressive, reckless, or self-destructive behaviors [10]. Emergency obstetrical interventions during labor and delivery can be a trigger for PTSD. A study published in 2021 [39] further supported this association and identified that emergency C-sections predicted higher post-traumatic stress symptoms than elective C-sections. PTSD is highly associated with PPD. Among women with PTSD, 65% also present with PPD, and conversely, 22% of women with PPD present with PTSD [6]. Because a negative perception of the childbirth experience is associated with the development of PTSD, emergency obstetrical interventions could explain the observed association between C-section and PPD symptoms [36].

### 2.5. Other Predictors of PPD

A prospective cross-sectional study identified low subjective social status, multiparity, low social support, and low mental health function as predictors significantly associated with PPD [16]. Others found primiparity to be a risk factor for developing PPD [27,32]. Socioeconomic factors such as low income and education have also been associated with an increased incidence of PPD [11]. Further, the link between a history of depression, including during pregnancy, and PPD symptoms is established [29,32]. Prenatal depression is classified as MDD with a peripartum onset that occurs during pregnancy [10].

### 2.6. Objectives

Therefore, to address the mixed findings linking C-sections to PPD symptoms, this study investigated: (1) the association between C-section type (planned vs. unplanned/emergency) and PPD, and (2) whether postpartum PTSD symptoms mediated this association. We hypothesized that compared to planned C-sections, unplanned C-sections would be associated with increased PPD symptoms and that the association between C-section delivery type and PPD symptoms would be mediated by PTSD symptoms.

## 3. Materials and Methods

### 3.1. Study Design and Sample

A secondary analysis of data from the Alberta Pregnancy Outcomes and Nutrition (APrON) longitudinal cohort study was performed. The APrON study design and methodological details are published elsewhere [40,41]. The primary purpose of the APrON study was to collect data on maternal and child mental health and nutrition and child neurodevelopment. The women recruited to the APrON study were a community sample of pregnant women living in two major cities in Alberta, Canada. Recruitment differed between the two cities as they have different approaches to prenatal and delivery care. In Calgary, research assistants were positioned in waiting rooms of maternity care and ultrasound clinics [40]. In Edmonton, recruitment was conducted in collaboration with the Women and Children’s Health Research Institute at the University of Alberta, which distributed information to the city’s main maternity clinics [40]. Women were eligible to participate in the study if they were older than 16 years of age and less than 27 weeks gestation at enrollment. Women unable to complete the questionnaire in English or who planned to move away from the study region within six months of enrollment were not eligible. The data that were used in this study were collected between May 2009 and May 2013. For this secondary analysis, the analytic sample was constrained to 354 mothers who gave birth via C-section. The criteria for inclusion in the current analysis were singleton delivery via C-section, with complete data on the study measures (i.e., EPDS and Psychiatric Diagnostic Screening Questionnaire (PDSQ)) at three months postpartum. Multiple gestation pregnancies were excluded from the study. Participants provided informed consent before study participation. The University of Calgary Health Research Ethics Board and the University of Alberta Health Research Ethics Biomedical Panel provided ethics approval (REB14-1702_REN7).

### 3.2. Measures

Data for this secondary analysis were collected in each trimester of pregnancy and at three months postpartum. Maternal demographic characteristics, obstetrical, and medical history data were obtained from prenatal and labor and delivery medical records.

#### 3.2.1. Predictor: C-Section Type

Labor and delivery medical records were used to determine the mode of delivery and C-section type. For this study, a variable was created whereby a C-section was coded 1 if emergency or unplanned and 0 if planned.

#### 3.2.2. Outcome: PPD Symptoms

PPD symptoms were measured at three months postpartum. The EPDS is a 10-question uni-dimensional self-report scale that uses a 4-point Likert scale with response categories ranging from 0 to 3 according to increasing severity of symptom [17]. The scores can range from 0 to 30. Previous research has revealed that the sensitivity of the EPDS for true positives for postpartum depressed women is 86%, and specificity for true negatives for non-depressed women is 78% [17]. A threshold score of greater than 12 represents women likely suffering from a major depressive illness [17]. A score of 9/10 results in failed detection of cases of under 10% and is an appropriate cutoff for use in primary care [17]. The EPDS has a standardized alpha coefficient of 0.87 and split-half reliability of 0.88 [17]. This tool has been translated into over 60 languages and is widely used internationally in clinical and research settings [42]. The EPDS scores were used as a continuous variable in this study.

#### 3.2.3. Mediator: PTSD Symptoms

The mediating variable of PTSD symptoms was assessed at three months postpartum using the PDSQ sub-scale for PTSD [43]. The PDSQ is a psychometrically strong self-report scale with a 15-item subscale for PTSD. Items are scored as 0 for “no” and 1 for “yes”. The scores can range from 0 to 15. The PTSD subscale has a Cronbach alpha of 0.94, and 92% sensitivity; a cut-off score of five or more represents the presence of PTSD symptoms [43]. The PDSQ has been used in the prenatal and postpartum period with PTSD prevalence rates aligning with rates in the literature [44].

#### 3.2.4. Covariates

Data used to estimate socioeconomic status (SES) were gathered at the first study visit and included mothers’ self-reported education, income, and occupation. This information was re-collected at three months postpartum. Maternal prenatal depression was also measured at each trimester using the EPDS scale, which has a positive predictive value of 80–88% and a negative predictive value of 94–95% in the prenatal period [45]. On the EPDS, the highest value across the first, second, or third trimester of pregnancy for each individual was used in the analyses. Data on the sex of the child and gestational age were obtained through a review of labor and delivery records. Social support was assessed in the first and second trimesters (not measured in the third trimester to reduce participant burden), using the Social Support Questionnaire [46], a four-item measure with scores ranging from 0 to 16 with higher values indicating greater perceived social support. The highest value for the first or second trimester was used in the analyses.

### 3.3. Data Analyses

Descriptive statistics were used to summarize participants’ sociodemographic characteristics. The relationships between the outcome and explanatory variables were examined using bivariate analysis. To test the hypotheses, Hayes conditional process modeling [47] was employed, using a linear regression-based PROCESS macro (5000 bootstraps and 95% CI) in SPSS version 24 from IBM (New York, NY, USA) (Hayes, 2013). The variables of socioeconomic status, social support, prenatal depression symptoms, PPD symptoms, and PTSD symptoms were used in the adjusted and unadjusted models. A priori power analysis was conducted using G*Power software version 3.1.9.7 (Heinrich Heine Universitat, Dusseldorf, Germany) for linear multiple regression models with fixed effects [48]. Given alpha = 0.05, an effect size [f^2^ = 0.07] derived from data showing a significant association between mode of delivery and PPD [28] our sample provides power > 0.8, including 6 predictors in each model. There was no missing data related to the main predictors and outcome variables. This enabled testing of both the direct and/or indirect effects of C-section type on PPD symptoms. Unadjusted and adjusted models were run, and covariates for the adjusted model were selected based on a combination of theoretical importance and bivariate correlations (*p* < 0.05).

## 4. Results

### 4.1. Descriptive Findings

Table 1 presents descriptive data on the sample. The mean age of mothers was 32.8 years, and the majority were married (98.2%), had post-secondary education (74.20%), had a household income ≥ $70,000 (86.46%) and were first-time mothers (62.5%). Over 50% had an unplanned or emergency C-section (*n* = 198; 55.93%). More of the children were male (54%).

Bivariate correlations were conducted between the study variables (see Table 2). PPD symptoms were significantly associated with SES, prenatal depression symptoms, and social support. PTSD symptoms were significantly associated with PPD symptoms as well as SES, prenatal depression symptoms, and social support.

### 4.2. Association between C-Section Type and PPD

**Figure 1:**
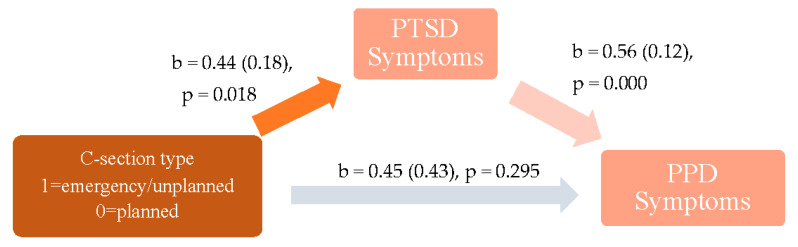
Unadjusted model for the association between C-section type (i.e., emergency C-section to planned C-section) and PTSD and PPD symptoms.

**Figure 2:**
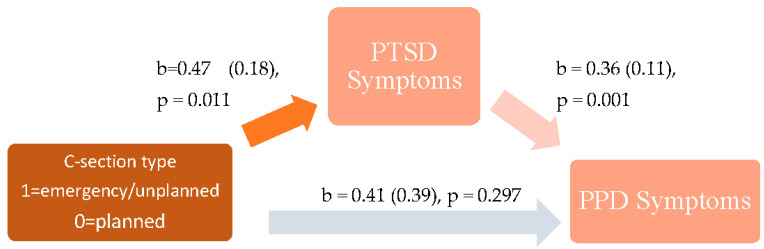
Adjusted model for the association between C-section type (i.e., emergency C-section compared to planned C-section) on PTSD and PPD symptoms.

The direct effect of C-section type on PPD symptoms was non-significant, in the unadjusted (β = 0.45 (*SE* = 0.43), 95% CI [−0.39, 1.30], *p* = 0.295) and adjusted (β = 0.41 (*SE* = 0.39), 95% CI [−0.36, 1.17], *p* = 0.297) models. See Figure 1 and Figure 2. This indicates that there was not a direct association between type of C-section (i.e., emergency/unplanned versus planned C-section) and mothers’ reported symptoms of PPD when their infants were three months old.

### 4.3. Mediation by Maternal Postnatal PTSD Symptoms

Examination of the unadjusted indirect effect of C-section type on PPD symptoms revealed a positive association between C-section type and PTSD symptoms (β = 0.44 (*SE* = 0.19) 95% CI [0.07, 0.80], *p* = 0.018) and PTSD and PPD (β = 0.56 (*SE* = 0.12), 95% CI [0.32, 0.81], *p* = 0.000). This suggests that mothers who experienced an emergency or unplanned C-section had increased PTSD scores by nearly half a point (0.44) compared to mothers who underwent a planned C-section. Further, higher PTSD scores were associated with higher PPD symptoms at three months postpartum. Specifically, for every one-unit increase in PTSD scores, PPD scores increased by 0.56 a point. The bootstrapped indirect effect of C-section type on PPD symptoms through PTSD symptoms as a mediator was significant, β = 0.25, [95% CI = 0.04, 0.52], suggesting that the effect of C-section on PPD symptoms is maybe via PTSD symptoms. This postpartum mediator model accounted for a 25.1% variance in PPD symptom scores at three months postpartum.

The same regression analyses were conducted to determine whether the effect of C-section type on PPD symptoms was mediated by PTSD symptoms, this time adjusting for prenatal depression symptoms, SES, and perinatal support. The direct effect of C-section type on PPD symptoms remained non-significant, (β = 0.41 (*SE* = 0.39), 95% CI [−0.36, 1.17], *p* = 0.297). Prenatal depression symptoms (β = 0.08 (*SE* = 0.02), 95% CI [0.03, 0.12], *p* = 0.001), C-section type (β = 0.47 (*SE* = 0.18),95% CI [0.11, 0.83], *p* = 0.011) and prenatal social support (β = −0.08 (*SE* = 0.04), 95% CI [−0.17, −0.00], *p* = 0.042) showed positive associations with PTSD symptoms. Furthermore, PTSD symptoms (β = 0.36 (*SE* = 0.11), 95% CI [0.14, 0.58], *p* = 0.001) and prenatal depression symptoms (β = 0.45 (*SE* = 0.05), 95% CI [0.35, 0.54] *p* = 0.000) also showed positive associations with PPD symptoms. The bootstrapped indirect effect of emergency C-section on PPD symptoms with PTSD symptoms as a mediator remained significant after controlling for prenatal depression symptoms, social support, and SES, (β = 0.17 (*SE* = 0.11), 95% CI [0.03, 0.42]). This suggests that mothers who experienced an emergency or unplanned C-section had increased PTSD scores of nearly half a point (0.47) compared to mothers who underwent a planned C-section, even after adjustment for prenatal depression symptoms, social support, and SES, and this was associated with an increase in PPD scores of 0.36 of a point. The total effect is not significant (β = 0.70 (*SE* = 0.44), 95% CI [−0.17, 1.57], *p* = 0.113).

## 5. Discussion

To the best of our knowledge, this study was the first to examine the association between C-section type and PPD symptoms and whether maternal PTSD symptoms mediated this association. No direct association was found between C-section type and PPD symptoms. However, emergency C-section was found to be indirectly associated with PPD symptoms via PTSD symptoms. The association remained significant after controlling for prenatal depression symptoms, SES, and social support. These findings suggest that unplanned operative deliveries may be associated with the development of PTSD symptoms in mothers, which could subsequently lead to the development of PPD symptoms.

Our findings support previous studies that have identified obstetrical interventions as a significant influential factor in the development of birth-related trauma symptoms, which are linked to an increased risk of PTSD and PPD [49,50]. Subjective childbirth experience is an important predictor of postpartum PTSD and is influenced by obstetric complications [51,52]. In our study, PTSD symptoms accounted for 25.1% of the variance in PPD symptoms at three months postpartum supporting a high association between PTSD and PPD symptoms. The correlation between PPD and PTSD is consistently supported in the literature [36,53,54,55]. PTSD symptoms among new mothers can result in feelings of rejection and avoidance as well as hypervigilance and anxiousness toward the infant [49], resulting in disordered maternal-child relationships [56]. Altered maternal-child relationships are also noted among women suffering from PPD, further supporting the co-occurring relationship between PPD and PTSD symptoms. Given the co-occurrence of PTSD and PDD, postpartum mental health screening should include PTSD screening. Universal screening for PTSD and PPD symptoms is essential if healthcare providers are to ensure appropriate treatment and follow-up and decrease the stigma surrounding postpartum mental health conditions [36,57]. The findings of this study provide support for the frequent co-occurrence of PTSD and PPD and provide validation for the supposition that PTSD screening should be considered in the assessment and provision of maternal mental health care [57].

The link identified between emergency C-sections and increased PTSD scores is further supported by recent studies evaluating mode of delivery and childbirth-related PTSD. A study performed in 2019, demonstrated that women who experienced an unplanned C-section had higher PTSD symptoms (M = 30.77, SD = 19.74) at three months postpartum as compared to women who experienced a planned C-section (M = 23.01, SD = 16.89) or vaginal delivery (M = 19.27, SD = 17.23) [50]. The association is further supported by a study of 166 mothers who delivered via C-section in Greece; the study identified that 31.7% of women who delivered via emergency C-section presented with PTSD versus 1% of women who had a planned C-section delivery [58]. The strong link between an emergency C-section and PTSD symptoms demonstrates the importance of understanding the trajectories of birth-related PTSD and how they intersect with PPD symptoms.

C-section delivery is a predictor of PPD symptoms [11,30,31]. Obstetrical complications such as hyperemesis gravidarum, gestational hypertension, pre-eclampsia, and C-sections have been linked to an increased risk of PPD [11]. Although our study did not identify this direct relationship, we established that an emergency C-section compared to a planned C-section is indirectly associated with PPD symptoms. A review failed to establish a link between C-section and PPD [14]; however, it was hypothesized that C-section may operate as a risk factor for women vulnerable to PPD such as those with negative labor experiences [14]. Given the rate of PPD among those with PTSD, postpartum PTSD may make women vulnerable to PPD symptoms. Our study may offer a pathway to understanding the association identified by previous studies. Negative birth experiences affect a woman’s perception of her birth and are linked to PTSD [36], which in turn is related to PPD [59]. Emergency C-section is the strongest factor associated with a negative evaluation of the birth experience at one year [33]. A systematic review reported that 11 of 15 studies identified a significant association between women’s perspectives of their birth experience and PPD symptoms [3]. Delivery via C-section or vaginal instrumental delivery was associated with higher somatization, depression, and overall distress [50], this further outlines the important effect of the mode of delivery on postpartum wellbeing. Knowledge regarding birth experience factors that contribute to the development of PPD will assist healthcare practitioners in identifying women at risk.

A history of depression is associated with an increased risk of PPD [27]. A study published in 2017 [27] reported that a previous history of depression is associated with the highest relative risk level (OR, 16.72; 95% CI, 16.05–17.42) for developing depression in the postpartum period. The relationship between prenatal and postnatal depression may explain the association between mode of delivery and PPD symptoms. Consistent with this, a longitudinal study of 753 [30] women reported an association between mode of delivery and PPD symptoms on the EPDS. This study also noted a significant relationship between prenatal EPDS values and delivery mode, with women undergoing a primary C-section having significantly higher prenatal depression scores.

While our findings are consistent with some previous research, they do not support other studies [16,32,60], which did not find an association between mode of delivery and PPD. This could be due to the timing of the assessment of PPD symptoms. For example, previous studies [16,60] measured the outcome of postnatal depression at six weeks and eight weeks postpartum, respectively. Further, assessment of PPD symptoms in the first weeks postpartum may be measuring postpartum blues rather than true PPD symptoms. Postpartum blues are characterized by symptoms of crying, irritability, anxiety, and restlessness that last one to two weeks after delivery [9]. A study performed [32] failed to identify an association between mode of birth and postnatal psychological morbidity at seven-ten days postpartum. Future research evaluating the association between mode of delivery and PPD symptoms should consider the timing of PPD symptoms assessment and differentiation for the type of C-section.

Consideration must be given to factors that put women at risk of altered postpartum mental health and risk factors for PPD symptoms. Antenatal healthcare providers need to be aware of factors that are associated with PPD symptoms. During pregnancy, care providers should screen women for depression because of the established relationship between prenatal depression and mode of delivery and subsequent PPD symptoms. Perinatal depression screening and treatment have been shown to decrease the risk of depression [61]. Universal PPD depression screening will potentially identify women at risk of developing PPD, enabling care providers to link mothers to timely and appropriate treatment [62]. Perinatal healthcare providers should also employ caring and effective communication strategies while being proactive in preventing childbirth-related trauma [63]. Communication strategies that may limit the development of childbirth trauma require an open dialogue with the patient regarding labor and delivery events occurring, a thorough history addressing particular fears, and determining if previous deliveries were perceived as traumatic [63]. Healthcare practitioners that provide prenatal, labor and delivery, and postpartum care ought to be knowledgeable about the predictors of PTSD and PPD symptoms so they can be alert to high-risk women. Additional care considerations should address modifiable risk factors associated with C-section delivery such as maternal request, practice variations among hospitals and healthcare providers, body mass index > 40, smoking status, and multiple gestation pregnancy [64,65,66].

## 6. Strengths and Limitations

The prospective longitudinal nature of the study design is a significant strength as it allowed for follow-up of participants from pregnancy through to three months postpartum. The study employed a community sample and employed well-validated measures for PTSD and PPD. The sample size allowed for relevant covariates to be controlled for in the analysis, while maintaining adequate power to support findings. However, a limitation is that the findings may be limited due to the use of a community sample and not a clinical sample. The sample under-represents women in lower education and income categories, as the mothers in the APrON study were primarily university-educated and of high income, impacting the generalizability of the results. Given that the initial study was initiated 12 years ago. This may be a limitation as prevalence rates for C-sections, PPD, and PTSD may be misrepresented. The use of maternal self-reports for both PTSD and PPD may also be a limitation, as is the collection of the mediator data at the same time as the outcome data. The use of the PDSQ may not be the most appropriate measurement tool for PTSD as its use has not been thoroughly validated among the perinatal population. Our study did not consider factors such as prior medication history, a history of intimate partner violence, or the circumstances surrounding pregnancy which may be pertinent given the topic of maternal mental health. Additionally, we controlled for the covariates of prenatal depression, SES, and social support; however, including data on mental health history, indication for C-section, and neonatal status such as neonatal intensive care admission and Apgar score may be beneficial to consider in future modeling.

## 7. Conclusions

This study provides evidence that unplanned, or emergency C-sections may play a role in the development of maternal PPD. The study found that emergency C-section was associated with more postpartum PTSD symptoms, which in turn was associated with a higher number of symptoms of PPD among new mothers. Overall, the current study demonstrates that emergency C-sections are indirectly associated with maternal PPD symptoms. These findings suggest that unplanned operative deliveries may have a negative effect on birth perceptions and subsequently could be associated with the development of PPD. Increased knowledge regarding factors contributing to the development of PTSD and PPD could assist healthcare practitioners in identifying women at risk for poor mental health in the postpartum period.

## Figures and Tables

**Table 1:** Socio-demographic and descriptive characteristics of study participants *n* = 345.

Variable	Frequency (*n*)	Percentage
Sociodemographic Maternal education		
Below university degree	89	25.80
University degree or more	256	74.20
Marital status		
Single	7	1.98
Married	347	98.02
Household Income		
Less than 70 k	47	13.54
70 k or more	300	86.46
	Mean (SD)	Range
Maternal age	32.80 (4.36)	23–44
Medical Characteristics		
Parity		
0	220	62.15
1	103	29.10
2	31	8.76
C-section Type		
Emergency/unplanned	198	55.93
Planned	156	44.07
Gestational age at birth		
<37 weeks	31	8.76
37 and more weeks	323	91.24
Child sex		
Male	192	54.24
Female	162	45.76
Psychological Measurements PTSD score		
5 or more (cutoff)	14	3.96
EPDS score (postpartum)	48	13.6
10 or more (cutoff)	Mean (SD)	Range
Prenatal depression symptoms	7.03 (4.13)	0–22
PPD symptoms	4.92 (4.12)	0–30
Prenatal social support	14.80 (2.22)	4–16
PTSD symptoms	0.64 (1.73)	0–13

**Table 2:** Bivariate correlations between demographics and pre-post maternal distress variables.

Variables	1	2	3	4	5	6
Socio-economic status (1)	-	−0.07 *	−0.21 **	0.11 **	−0.11 **	−0.08 *
Parity (2)		-	0.01	−0.13 **	−0.00	−0.01
Prenatal depression symptoms (3)			-	−0.28 **	0.22 **	0.54 **
Prenatal social support (4)				-	−0.16 **	−0.20 **
PTSD symptoms (5)					-	0.27 **
PPD symptoms (6)						-

Note * *p* < 0.05, ** *p* < 0.01.

## Data Availability

To access this data, please email APrON Principal Investigator, Nicole Letourneau at Nicole.Letourneau@ucalgary.ca. Data may also be accessed via the PolicyWise Secondary Analysis to Generate Evidence (SAGE) repository (https://policywise.com/sage/ (accessed on 15 January 2021)).
